# Absorbed plant MIR2911 in honeysuckle decoction inhibits SARS-CoV-2 replication and accelerates the negative conversion of infected patients

**DOI:** 10.1038/s41421-020-00197-3

**Published:** 2020-08-05

**Authors:** Li-Kun Zhou, Zhen Zhou, Xia-Ming Jiang, Yishan Zheng, Xi Chen, Zheng Fu, Gengfu Xiao, Chen-Yu Zhang, Lei-Ke Zhang, Yongxiang Yi

**Affiliations:** 1 Nanjing Drum Tower Hospital Center of Molecular Diagnostic and Therapy, State Key Laboratory of Pharmaceutical Biotechnology, Jiangsu Engineering Research Center for MicroRNA Biology and Biotechnology, NJU Advanced Institute of Life Sciences (NAILS), NJU Institute of AI Biomedicine and Biotechnology, School of Life Sciences, Nanjing University, Nanjing, Jiangsu 210023 China; 2 Tianjin Medical University Cancer Institute and Hospital, National Clinical Research Center for Cancer, Tianjin’s Clinical Research Center for Cancer, Key Laboratory of Cancer Prevention and Therapy, Tianjin, 300060 China; 3 State Key Laboratory of Virology, Wuhan Institute of Virology, Center for Biosafety Mega-Science, Chinese Academy of Sciences, Wuhan, Hubei 430071 China; 4 Department of critical Care Medicine and Nanjing infectious Disease Center, the Second Hospital of Nanjing, Nanjing University of Chinese Medicine, Nanjing, Jiangsu 210003 China

**Keywords:** miRNAs, DNA synthesis

Dear Editor,

The Coronavirus disease 2019 (COVID-19) pandemic is one of the most serious global public health crises to date. As of July 12, 2020, more than 12.6 million cases of COVID-19 infection with 0.56 million deaths were confirmed worldwide^1^. Since there are no effective therapeutics to treat severe acute respiratory syndrome coronavirus 2 (SARS-CoV-2, the causative virus of COVID-19) infection so far, the pandemic is rapidly spreading worldwide. It is urgent to develop effective therapies, not only to treat infected patients but also to control the pandemic.

Our previous studies have demonstrated that a plant microRNA, MIR2911, which is enriched in honeysuckle decoction (HD), directly targets influenza A viruses (IAV), including H1N1, H5N1, and H7N9 subtypes by binding to their mRNA and blocking protein translation. Oral administration of HD can prevent IAV infection and reduce H5N1-induced mouse death^2^. Subsequent studies have shown that MIR2911 also directly inhibits the replication of various viruses in addition to IAVs^3,4^. Upon dietary uptake, these microRNAs self-assemble into exosomes and are then secreted into the circulation and delivered into target tissues or specific cells, including the liver, lung, spleen, pancreas, and T cells^5–7^. Given the unique GC-enriched nucleotide composition of MIR2911 (GGCCGGGGGACGGACUGGGA) and after we analyzed the genome sequence of SARS-CoV-2, it is most likely that the virus genome contains MIR2911-binding sites and that MIR2911 can inhibit SARS-CoV-2 replication directly. In the present study, we assessed the inhibitory effect of absorbed MIR2911 in HD on SARS-CoV-2 replication and conducted a clinical study to investigate the efficacy of HD in COVID-19 patients.

By using bioinformatics analysis, we predicted that there are 179 putative MIR2911-binding sites in the SARS-CoV-2 genome. Twenty-eight binding sites (Supplementary Table S1) were confirmed by classic luciferase assay (Supplementary Fig. S1), which are distributed widely in the virus genome (Fig. 1a), indicating that MIR2911 may be able to inhibit the translation of almost all the proteins of SARS-CoV-2.

**Fig. 1:**
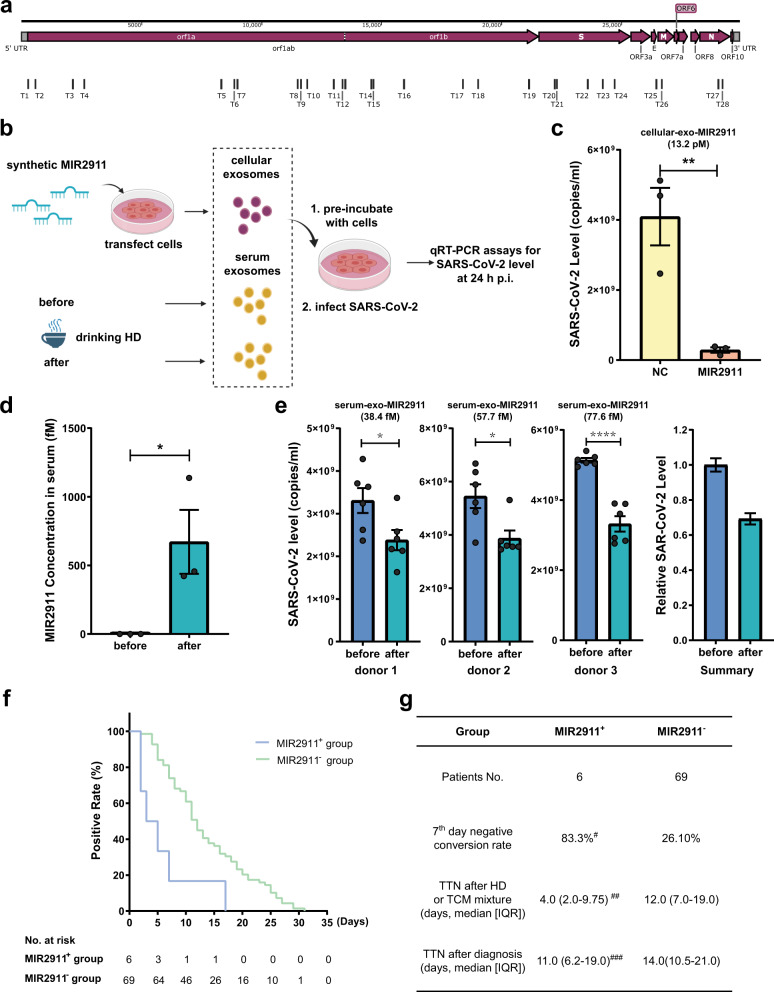
Absorbed MIR2911 in HD directly inhibits SARS-CoV-2 replication. **a** Diagrams of MIR2911-binding sites on SARS-CoV-2 genome. **b** Diagrams of the collection of exosomes from cell medium or serum before or after the oral administration of HD and the measurement of the antiviral activities of cellular or serum exosomal miRNAs. **c** Efficacy of exosomes secreted by HEK293T cells transfected with MIR2911 or ncRNA in inhibiting replication of SARS-CoV-2. Vero E6 cells were pretreated with cellular exosomes for 8 h, and virus was then added to incubate with cells at an MOI of 0.01 for 1 h. Afterwards, exosomes and virus mixture was removed and the cells were cultured with fresh medium until the end of the experiment. Virus yield in the infected cell supernatants was quantified by qRT-PCR (*n* = 3) at 24 h p.i. **d**, **e** Efficiency of exosomal-MIR2911 in human serum to inhibit replication of SARS-CoV-2. Serum exosomes were harvest from three donors before and 2 h after oral administration of HD (10.5 pmol MIR2911/200 ml, once/30 g dry honeysuckle/per donor). MIR2911 concentration in serum were measured by qRT-PCR (**d**). Vero E6 cells were pretreated with human serum exosomes for 8 h, and virus was then added to incubate with cells at an MOI of 0.01 for 1 h. Afterwards, exosomes and virus mixture was removed and the cells were cultured with fresh medium until the end of the experiment. Virus yield in the infected cell supernatants was quantified by qRT-PCR (**e**, *n* = 6) at 24 h p.i. **f**, **g** Comparison of TTN (**f**, **g**) and 7th day negative conversion rate (**g**) between MIR2911^+^ group and MIR2911^−^ group. MIR2911^+^ group took HD (10.5 pmol MIR2911/oral 100 mL, twice daily/30 g dry honeysuckle/per patient, 600 ml water boiled with 30 g dry honeysuckle for 80 min until obtaining 200 ml HD). MIR2911^–^ group took traditional TCM orally. All included patients are moderate type. MIR2911^+^ group patients were from a clinic study conducted at Nanjing second hospital. MIR2911^–^ group patients were from the same hospital. Seventh day negative conversion rate defined as the percentage of patients converted to SARS-CoV-2 PCR-negative at the 7th day. The day patients received first dose of HD or TCM mixture was recorded as 1st day. Viral negative results must be confirmed twice of 24-h interval. The data are expressed as means ± SEM. *P < 0.05, ****P < 0.0001 using two-tailed Student’s *t* test; ^#^*P* = 0.004 compared with MIR2911^−^ group using *χ*^2^ test; ^##^*P* = 0.005 compared with MIR2911^−^ group (HR 0.28, 95% CI 0.12–0.67) using Cox regression with the adjustment of sex factor; ^###^*P* > 0.05 compared with MIR2911^−^ group (HR 0.61, 95% CI 0.68–3.94) using Cox regression with the adjustment of sex factor. HD honeysuckle decoction, TTN time taken to become SARS-CoV-2 PCR-negative, TCM traditional Chinese medicine, IQR interquartile range, HR Hazard ratios, CI confidence intervals.

In order to assess the direct effect of absorbed MIR2911 on SARS-CoV-2 replication, cellular exosomes were collected from culture medium of HEK293T cells transfected with synthetic MIR2911 or control non-coding RNA (ncRNA), as similar method to previous report^5^ (Fig. 1b). Isolated cellular exosomes with/without MIR2911 were separately pre-incubated with 5 × 10^4^ Vero E6 cells (ATCC-1586) in 0.25 ml cell medium for 8 h. After changing the culture medium, the cells were infected with SARS-CoV-2 (nCoV-2019BetaCoV/Wuhan/WIV04/2019^8^) at a multiplicity of infection (MOI) of 0.01. Efficacies were evaluated by quantification of viral copy numbers in the cell supernatant via quantitative real-time RT-PCR (qRT-PCR) at 24 h post infection (p.i.) (Fig. 1b). As shown in Fig. 1c, cellular exosomal-MIR2911 at 13.2 pM concentration inhibited 93% virus replication (from 4.09 × 10^9^ to 2.87 × 10^8^ copies/ml), indicating that absorbed MIR2911 directly and sufficiently inhibits SARS-CoV-2 replication (Fig. 1c). Next, we assessed the inhibitory effect of absorbed MIR2911 in HD on SARS-CoV-2 replication. Since absorbed MIR2911 in HD is delivered into the lung by exosomes through circulation, isolated exosomes from 62.5 μl of donor serum before and after drinking 200 ml HD (30 g dry honeysuckle) separately were pre-incubated with 5 × 10^4^ Vero E6 cells, as similar condition to that of cellular exosomal-MIR2911 (Fig. 1b). The MIR2911 concentration in 200 ml HD was 52.5 pM (10.5 pmol/200 ml/30 g dried honeysuckle). Serum levels of MIR2911 in three heathy receivers after drinking for 2 h were 0.42, 0.45, and 1.13 pM (Fig. 1d), which were undetectable before drinking). Exosomes (MIR2911 levels: before drinking, nondetectable; after drinking, they were 9.9, 14.4, and 19.4 amol) were pre-incubated with 5 × 10^4^ cells (Fig. 1e, ~180 copies of MIR2911/cell). As shown in Fig. 1e, exosomes containing MIR2911 significantly inhibited virus replication (Fig. 1e). There is no difference of cell viabilities between exosomes with/without MIR2911 collected from the same donor before and after drinking HD (Supplementary Fig. S2). These results strongly suggest that MIR2911 is sufficiently absorbed by consumers drinking HD and that the absorbed MIR2911 significantly blocks SARS-CoV-2 replication.

To assess the antiviral effect of MIR2911 in HD on COVID-19 patients, we conducted a clinical study. Seventy-five moderate type COVID-19 patients who received routine antiviral therapy (RT) at Nanjing Second Hospital from January 2020 to March 2020 were enrolled in this study. Patients were divided into two groups based on additional treatment with MIR2911 in HD (MIR2911^+^) or traditional Chinese medicine (TCM) mixture (MIR2911^–^) in addition to RT. The primary endpoint was the negative conversion rate on the 7th day from the first treatment. There were 6 and 69 patients in the MIR2911^+^ and MIR2911^–^ groups, respectively (Supplementary Tables S2–S4). The negative conversion rate on the 7th day in the MIR2911^+^ group was 83.3%, which was dramatically improved compared with that of patients treated with MIR2911^–^ TCM (26.1%, *P* = 0.004) (Fig. 1g, f). The time taken to become SARS-CoV-2 PCR-negative (TTN) also favored patients treated with HD-MIR2911 (median 4.0 vs. 12.0 days, HR 0.28, 95% CI 0.12–0.67, *P* = 0.005) (Fig. 1g, f). The median TTN of male patients in MIR2911^+^ (1 case) and MIR2911^–^ (38 cases) are 5.0 days and 11.0 days (HR 0.003, 95% CI 0.000018–0.52, *P* = 0.027), respectively. The median TTN of female patients in MIR2911^+^ (5 cases) and MIR2911^–^ (31 cases) groups are 3.0 days and 12.0 days (HR 0.15, 95% CI 0.031–0.68, *P* = 0.014), respectively.

HD has been used to treat viral infections for a thousand years in China. Previous studies have demonstrated that MIR2911 (0.06–0.18 pmol/day) in 1–3 ml HD significantly inhibits influenza virus replication in 20 g mouse^2^. In addition to the Pharmacopoeia of the People’s Republic of China^9^, we chose 30 g dried honeysuckle (MIR 2911 level: 10.5 pmol) per day for use. Our results demonstrate that 30 g dried honeysuckle is safe for use (Supplementary Table S4) and has sufficient antiviral function (Fig. 1d, e). On the other hand, the data that MIR2911 (~60 fM) in exosomes significantly inhibits virus replication not only confirms the extra-high antiviral activity of MIR2911 (compared with that of remdesivir: 3.7 μM and Chloroquine: 10 μM)^10^ but also provides a novel strategy that using serum exosomes collected from healthy donor offers the most similar condition in vivo to assess the efficacy of potential drugs in vitro.

In summary, our results suggest that absorbed plant MIR2911 in HD inhibits SARS-CoV-2 replication and accelerates the negative conversion of infected patients. HD treatment might greatly help cure infected patients and stop the COVID-19 pandemic.

## Supplementary information

Supplementary information

## Supplementary information

**Supplementary Information** accompanies the paper at (10.1038/s41421-020-00197-3).
