# Supplementary material for: Absorbed plant MIR2911 in honeysuckle decoction inhibits SARS-CoV-2 replication and accelerates the negative conversion of infected patients
Source: Cell Discov. 2020 Aug 5;6:54. doi: 10.1038/s41421-020-00197-3 (PMC7406496; doi:10.1038/s41421-020-00197-3)
Supplement: Supplementary file 1 — Supplementary information [file 41421_2020_197_MOESM1_ESM.pdf]

Supplementary Information

Materials and Methods

Cells, virus and Honeysuckle

African green monkey kidney Vero E6 cell line was obtained from American Type Culture Collection (ATCC, no. 1586) and cultured in dulbecco's modified eagle medium (DMEM; Gibco) supplemented with 10% fetal bovine serum (FBS; Gibco) at 37 °C with 5% CO_2. HEK293T cells was obtained from Type Culture Collection of Chinese Academy of Sciences and cultured in high glucose DMEM (Gibco) supplemented with 10% FBS and 1% penicillin-streptomycin at 37 °C with 5% CO_2.

A clinical isolate SARS-CoV-2 (nCoV-2019BetaCoV/Wuhan/WIV04/2019[1]) was propagated in Vero E6 cells, and viral titer was determined by 50% tissue culture infective dose (TCID50). All experiments with live SARS-CoV-2 viruses were conducted within the biosafety level 3 (BSL3+) facility.

Dry honeysuckle was purchased from the local TCM store. For honeysuckle decoction, we boiled 30 g dry honeysuckle in 600 ml water for 80 minutes and got 200 ml herb decoction.

miRNA target prediction and Luciferase report assay

SARS-CoV-2 genome sequence was acquired from the NCBI database (Refseq ID: MN908947). RNAhybrid[2] was employed to predict potential MIR2911 binding sites on SARS-COV-2 genome.

To test the binding efficiency of MIR2911 to the SARS-CoV-2 genes, a plasmid carrying the luciferase gene linked to a fragment of the target gene 3'-UTR harboring putative MIR2911 binding sites was co-transfected into HEK293T cells along with synthetic NC or MIR2911 (GenePharm, China). HEK293T cells were cultured in DMEM containing 10% FBS and seeded in 24-well plates. At 24 h after plating, 0.2 μg of firefly luciferase reporter plasmid, 0.1 μg of β-galactosidase expression vector (Ambion, CA, USA) and equal amounts (20 pmol) of synthetic MIR2911 were transfected into cells with Lipofectamine 2000 (Invitrogen, CA, USA) according to the manufacturer's instructions. The β-galactosidase plasmid was used as a transfection control. At 24 h post-transfection, the cells were analysed using a luciferase assay kit (Promega, WI, USA). All experiments were performed in triplicate wells for each condition independently.

Human serum Sample Collection

To assess the function of exosomal MIR2911 in serum, this study enrolled 3 donors. Every donor was fed 200 ml honeysuckle decoction, which was prepared from 30 g dried honeysuckle. 10 ml

Serum samples were collected before and 2 hours after oral administrating honeysuckle decoction.

Exosome isolation

For exosome collection from culture cells, HEK293T cells were transfected with synthetic MIR2911 or ncRNA by using Lipofectomine 2000 (Invitrogen, US). After 36 h, exosomes were isolated from the cell culture medium using a Total Exosome Isolation Reagent (from cell culture medium, Invitrogen, US) according to the manufacturer's instructions. Briefly, after cell debris and shedding vesicles were removed by centrifugation at 2000×g for 30 min and then at 10000×g for 1 h, the supernatant was mixed with isolation reagent and incubated at 4°C overnight. The solution was centrifuged at 3000×g for 1 h (all steps were performed at 4°C). Exosome pellets were collected and re-suspended in PBS.

For human serum exosome collection, exosomes were isolated from human serum samples using a Serum Exosome Isolation Kit (Vazyme, China) according to the manufacturer's instructions. Exosome pellets were collected and re-suspended in DMEM.

RNA isolation and quantitative RT-PCR assays

Small RNAs (<100 nt) from honeysuckle decoction were extracted using the Universal Plant MicroRNA Kit (Biotech, China) according to manufacturer's instructions. Total RNAs from human exosome were extracted using TRIZOL reagent (Invitrogen) according to the manufacturer's instructions.

To detect MIR2911 level, quantitative RT-PCR was performed using TaqMan miRNA probes (Applied Biosystems, USA) using an LC96 PCR machine according to the manufacturer's instructions. A series of synthetic MIR2911 oligonucleotides at known concentrations was reverse transcribed and amplified to build standard curve. The absolute amount of MIR2911 was then calculated in reference to the standard curve.

Evaluation of antiviral activities of exosomes

The cell viabilities of serum exosomes on Vero E6 Cells were determined by CCK8 assays (GLPBio, USA). To test the antiviral efficacy of serum exosome from donors, Vero E6 cells were cultured overnight in 48-well cell-culture petridish with a density of 5×10^4 cells/well. Cells were pretreated with cellular exosomes isolated from 1.5 ml cell medium or serum exosomes isolated from 62.5 μl serum from different doners for 8 hours. Subsequently, treated Vero E6 cells were infected with SARS-CoV-2 at a multiplicity of infection (MOI) of 0.01. After 1 h of incubation, the virus-exosome mixture was removed and cells were washed with warm PBS and incubated in

fresh medium. At 24 hours p.i., the cell supernatant was collected and lysed. The viral RNA extraction and quantitative real time PCR (RT-PCR) analysis was described in our previous study[3].

Clinical Study

Clinical Study

Moderate type patients infected by SARS-CoV-2 virus in Nanjing Second Hospital from January 2020 to March 2020 were included to this study. As MIR2911 were abundant in honeysuckle decoction (10.5 pmol /30g honeysuckle) and undetectable in Traditional Chinese Medicine (TCM) mixture, patients were divided into two groups, the MIR2911^{+} group (patients received routine anti-viral therapy plus honeysuckle decoction,10.5pmol MIR2911/oral 100ml, twice daily/30g dry honeysuckle/per patient for 14 consecutive days) and MIR2911^{-} group (patients received routine anti-viral therapy plus TCM mixture). All 6 patients from a clinical study (Chinese Clinical Study Register number, ChiCTR2000029822) were enrolled in MIR2911^{+} group. Other 69 patients received anti-viral therapy plus TCM mixture treated at the same hospital were enrolled in MIR2911^{-} group.

All enrolled patients have pneumonia confirmed by chest computed tomography imaging. COVID-19 infection was determined by pharyngo swab quantitative RT-qPCR assay which performed by either local Chinese Center for Disease Control and Prevention (CDC) or the designated diagnostic laboratory in the hospital.

COVID-19 Diagnosis

RNA was extracted from pharyngo swab samples by nucleic acid isolation kit (magnetic beads) supplied by Bioperfectus Technologies. Quantitative RT-PCR was performed by ABI 7500 with Novel Coronavirus 2019-nCoV Nucleic Acid Testing Kit in accordance with the instructions of the manufacturer (BGI BIOTECHNOLOGY, WUHAN, Lot number:6020200217). RT-qPCR results can be reported as positive when it fulfilled either of the following criteria: 1. Ct value $\leq$38 with a "s" shape amplification curve; 2. Double testing amplification curves are both "s" shape if Ct value >38.

Outcome Measures

The primary endpoint was SARS-CoV-2 negative conversion rate at the 7th day, defined as the percentage of enrolled patients converted to SARS-CoV-2 PCR-negative at the 7th day. The day patients received first dose of honeysuckle decoction or TCM mixture was recorded as 1st day. The second outcomes were times taken to become SARS-CoV-2 PCR-negative after honeysuckle decoction/TCM mixture or since diagnosis as Covid-19 infection. Adverse events (AE) were classified according to the National Cancer Institute Common Terminology Criteria for Adverse Events, version 5.0(CTCAE 5.0).

Statistics

Data is presented as mean ± SEM. When comparing the antiviral effect and cell viabilities of exosomes from two groups, *P* values are calculated using two-tailed students' t test. When comparing TTN and HD-TTN of $MIR2911^{+}$ and $MIR2911^{-}$ groups, *P* values are calculated using Cox regression with the adjustment of sex factor. When comparing HD-TTN of male or female patients, *P* values are calculated using log-rank test and Mantel-Haenszel method.

References

1 Zhou, P. *et al.* A pneumonia outbreak associated with a new coronavirus of probable bat origin. *Nature* **579**, 270-273, doi:10.1038/s41586-020-2012-7 (2020).

2 Rehmsmeier, M., Steffen, P., Hochsmann, M. & Giegerich, R. Fast and effective prediction of microRNA/target duplexes. *RNA* **10**, 1507-1517, doi:10.1261/rna.5248604 (2004).

3 Wang, M. *et al.* Remdesivir and chloroquine effectively inhibit the recently emerged novel coronavirus (2019-nCoV) in vitro. *Cell Res* **30**, 269-271, doi:10.1038/s41422-020-0282-0 (2020).

Supplementary information, Fig. S1

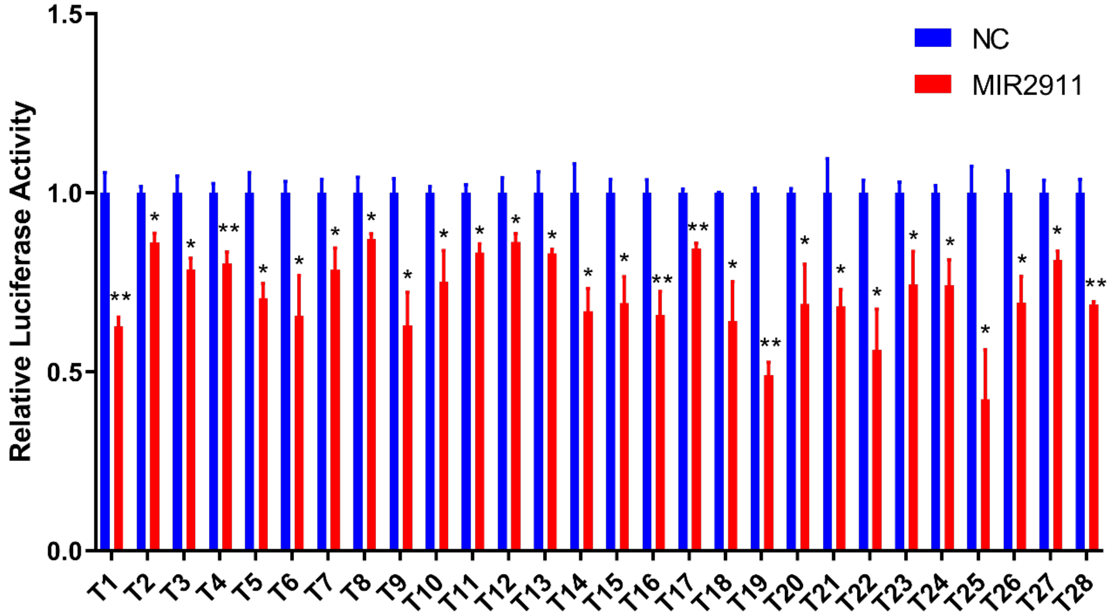

Fig. S1 Luciferase activity assays of HEK293T cells co-transfected with firefly luciferase reporters containing binding site sequences of MIR2911 in SARS-CoV-2 genome and MIR2911 or NC miRNAs.

Supplementary information, Figure S2

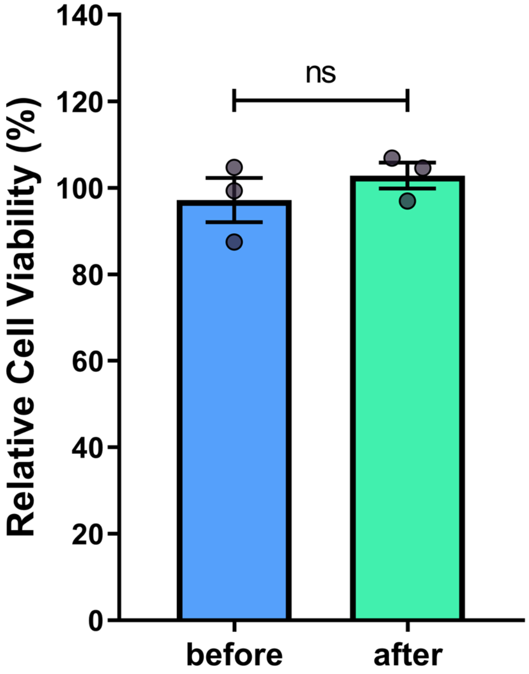

Fig. S2 CCK8 assay of Vero E6 cells treated with donors' serum exosomes before or after the oral administration of HD.

Table S1 MIR2911 binding sites on SARS-CoV-2 genome

ID	Position	MFE (kcal/mol)	Base-pair information		
T1	271	-22.4	target 5'	G UUG U	3'
				AG CC UCCCUGGUU	
				UC GG GGGGGCCGG	
			miRNA 3'	AGGG A CA	5'
T2	574	-26.2	target 5'	A GGU UU AUGUG G	3'
				CUU GUCC GUCCCUC GGC	
				GGG CAGG CAGGGGG CCG	
			miRNA 3'	A U G	5'
T3	2134	-27.8	target 5'	A AAC UGA U	3'
				CUCA CCGUCCU UUGGCU	
				GGGU GGCAGGG GGCCGG	
			miRNA 3'	A CA	5'
T4	2608	-26.4	target 5'	A UU AUUAA G U	3'
				CCAGU GU C GGCU	
				GGUCA CA G CCGG	
			miRNA 3'	AG GG GGG G	5'
T5	8315	-22.4	target 5'	A CC GA G	3'
				CA CCGU CCUUGGU	
				GU GGCA GGGGCCG	
			miRNA 3'	AGG CA G G	5'
T6	8863	-26.2	target 5'	G UU A	3'
				CCUGGU G CCUGGC	
				GGGUCA C GGGCCG	
			miRNA 3'	A GG AGG G	5'

T7	8995	-22.6	target 5’ A C G G 3'<br>UCAG UUGU UUUUGGCU<br>GGUC GGCA GGGGCCGG<br>miRNA 3’ AG A G 5'
T8	11502	-21.8	target 5’ G G UACAA AUGU A 3'<br>U UAGU CUGUC UUUUGGCC<br>G GUCA GGCAG GGGGCCGG<br>miRNA 3’ A G 5'
T9	11636	-20.8	target 5’ A U A U 3'<br>CU GUU CUUUGGCC<br>GG CAG GGGGCCGG<br>miRNA 3’ AG U GCAG 5'
T10	11674	-22.2	target 5’ C A A G 3'<br>UUUAG CUG CUCUUGGU<br>GGGUC GGC GGGGGCCG<br>miRNA 3’ A A A G 5'
T11	13007	-26.1	target 5’ G A A ACAAG A 3'<br>CC CAGU CGUCU CUGGU<br>GG GUCA GCAGG GGCCG<br>miRNA 3’ A G G G 5'
T12	13367	-26.6	target 5’ A ACCG G A 3'<br>CAGUCUGU UCU CGGU<br>GUCAGGCA GGG GCCG<br>miRNA 3’ AGG G G 5'
T13	13484	-27	target 5’ G G C UACA U G 3'<br>U CAG CCGUCU CCG GC<br>G GUC GGCAGG GGC CG<br>miRNA 3’ A G A G G 5'

T14	14570	-21.9	target 5’ A UGCUAU CACG A 3'<br>CCC G CUG CUUCUGGU<br>GGG C GGC GGGGGCCG<br>miRNA 3’ A U A A G 5'
T15	14658	-27.6	target 5’ U AA AAA A 3'<br>UUUCA CUGUC CCCGGU<br>AGGGU GGCAG GGGCCG<br>miRNA 3’ CA G G 5'
T16	15932	-29.2	target 5’ A A A AAGAA AGG G 3'<br>CCCAG UCC UC UCCU GGCC<br>GGGUC AGG AG GGGG CCGG<br>miRNA 3’ A C 5'
T17	18386	-25.7	target 5’ A UG A ACA A 3'<br>CCUAGU CUGU CCU GGUU<br>GGGUCA GGCA GGG CCGG<br>miRNA 3’ A G G 5'
T18	19015	-26.5	target 5’ U U ACGACA A 3'<br>UCCCAGUUC UC UUGGU<br>AGGGUCAGG AG GGCCG<br>miRNA 3’ C GG G 5'
T19	21138	-25.8	target 5’ G C UGGAG G A 3'<br>CUAG UCU GUUCC UGGCU<br>GGUC AGG CAGGG GCCGG<br>miRNA 3’ AG G 5'
T20	22211	-25.8	target 5’ C G U 3'<br>CUCAG GUUUUUCGGCU<br>GGGUC CAGGGGGCCGG<br>miRNA 3’ A AGG 5'

T21	22300	-21.7	target 5’ A AUU A G 3'<br>AGUU UG CUCCUGGU<br>UCAG GC GGGGGCCG<br>miRNA 3’ AGGG A G 5'
T22	23588	-27.0	target 5’ A A AAU U G 3'<br>CUCAG CU UC CCUCGGC<br>GGGUC GG AG GGGGCCG<br>miRNA 3’ A A C G 5'
T23	24210	-25.6	target 5’ C G A G 3'<br>UUCUGGUU G CCUUUGGU<br>AGGGUCAG C GGGGGCCG<br>miRNA 3’ G A G 5'
T24	24705	-30.1	target 5’ A U U A A 3'<br>UCUUA GUCC UCCCUC GUC<br>AGGGU CAGG AGGGGG CGG<br>miRNA 3’ C C 5'
T25	26444	-26.1	target 5’ C AGU UGA U 3'<br>UUCUAG UCC UCUUCUGGUC<br>AGGGUC AGG AGGGGGCCGG<br>miRNA 3’ C 5'
T26	26670	-25.5	target 5’ A AAUUU G 3'<br>AGUU UCCUCUGGCU<br>UCAG AGGGGGCCGG<br>miRNA 3’ AGGG GC 5'
T27	29027	-20.9	target 5’ G G GCU AAGAAG A 3'<br>CU AG UCU CCUCGGC<br>GG UC AGG GGGGCCG<br>miRNA 3’ A G CAG G

				5'
T28	29197	-26.7	target 5' C CGCU AG A<br>CCCAG UC CGUUCUUCGG<br>GGGUC AG GCAGGGGGCC<br>miRNA 3' A GG	3'<br><br><br>5'

Table S2 Characteristics of enrolled patients

	Total	MIR2911^{+} group*	MIR2911^{-} group*
	N=75	N=6	N=69
**Age, median (IQR) – year** (*P*=0.13)	43 (30.0-61.0)	41 (19.5-73.7)	43 (30.0-60.0)
**Hubei exposure** (*P*=0.68)	30 (41.1)	2 (40)	28 (41.2)
**Time to take HD or TCM mixture since diagnosis, median (IQR) – day** (*P*=0.19)	2.0 (1.0-3.0)	2.0 (1.5-12.5)	2.0 (1.0-3.0)
**Gender - no. (%)**			
Male (*P*=0.07)	39 (52.0)	1 (16.7)	38 (55.1)
Female (*P*=0.07)	36 (48.0)	5 (83.3)	31 (44.9)
**Initial symptoms - no. (%)**			
Fever (*P*=0.89)	27 (36.0)	2 (33.3)	25 (36.2)
Cough (*P*=0.22)	14 (18.7)	0	14 (20.3)
Fever and cough (*P*=0.22)	14 (18.7)	0	14 (20.3)
Other (*P*=0.41)	6 (8.0)	1 (20.0)	5 (7.2)
No (*P*=0.28)	13 (17.3)	2 (40.0)	11 (15.9%)
**Coexisting disease - no. (%)**			
Hypertension (*P*=0.09)	9 (12.0)	2 (40.0)	7 (10.1)
Diabetes (*P*=0.45)	6 (8.0)	0	6 (8.7)
HBV infection (*P*=0.67)	2 (2.7)	0	2 (2.9)
Thrombocytopenia (*P*=0.81)	1 (1.3)	0	1 (1.4)
Cerebrovascular disease (*P*=0.81)	1 (1.3)	0	1 (1.4)
**Other anti-viral agents - no. (%)**			
Interferonα	75 (100)	6 (100)	69 (100)
Lopinavir/Ritonavir (*P*=0.003)	60 (80.0)	2 (33.3)	58 (84.0)

Darunavir/ Cobicistat (P=0.01)	27 (36.0)	4 (66.7)	23 (33.3)
Arbidol Hydrochloride (P=0.41)	37 (49.3)	2 (33.3)	35 (50.7)
IVIG (P=0.18)	32 (42.7)	1 (16.7)	31 (44.9)
Ribavirin (P=0.41)	7 (9.3)	0 (0.0)	7 (10.1)
Chloroquine (P=0.50)	5 (6.7)	0 (0.0)	5 (7.2)
**Laboratory testing no. (%)**			
C-reactive protein < 10 ng/ml (P=0.88)	52 (69.3)	4 (66.7)	48 (69.5)
C-reactive protein ≥ 10 ng/ml (P=0.88)	23 (30.7)	2 (33.3)	21 (30.5)
White-cell count < 4 × 10^9/L (P=0.28)	28 (37.3)	1 (16.7)	27 (39.1)
White-cell count 4–10 ×10^9/L (P=0.25)	46 (61.3)	5 (83.3)	41 (59.4)
White-cell count > 10 ×10^9/L (P=0.76)	1 (1.4)	0 (0.0)	1 (1.5)
Lymphocyte count ≥0.8 ×10^9/L (P=0.28)	62 (82.6)	4 (66.7)	58 (84.1)
Lymphocyte count <0.8 ×10^9/L (P=0.28)	13 (17.4)	2 (33.3)	11 (15.9)
Platelet count ≥85 × 10^9/L (P=0.67)	73 (97.3)	6 (100.0)	67 (97.1)
Platelet count <85 × 10^9/L (P=0.67)	2 (2.7)	0 (0.0)	2 (2.9)
Procalcitonin < 0.051 ng/ml (P=0.34)	65(86.7)	6 (100.0)	59 (85.5)
Procalcitonin ≥ 0.051 ng/ml (P=0.34)	10 (13.3)	0 (0.0)	10 (14.5)

HD, denotes honeysuckle decoction; IQR, interquartile range; HBV, hepatitis B Virus; IVIG, intravenous immunoglobulin. The statistically difference of clinical characteristics between MIR2911$^+$ and MIR2911$^-$ groups was performed by chi square test.

Table S3 Characteristics and results of MIR2911^{+} group

Patient No.	Age	Gender	Disease severity	Hubei exposure	Initial symptoms	HD-TTN*(days)	TTN after diagnosis	SIDT1 polymorphism§	Other anti-viral agents
1	50	M	moderate	No	Fever	5	7	WT/WT†	Interferonα1b, Darunavir/Cobicistat Arbidol Hydrochloride
2	72	F	moderate	YES	No	2	5	WT/WT	Interferonα1b, Darunavir /Cobicistat
3	79	F	moderate	No	Fatigue	3	13	WT/WT	Interferonα1b, Darunavir/Cobicistat, Arbidol Hydrochloride, IVIG
4	9	F	moderate	No	Fever	7	9	WT/WT	Interferon2b
5	23	F	moderate	YES	No	2	22	WT/WT	Interferonα1b, Lopinavir/Ritonavir
6	32	F	moderate	No	No	18	18	WT/MUT‡	Interferonα1b, Lopinavir/Ritonavir Darunavir/Cobicistat, Arbidol Hydrochloride**

* HD-TTN denotes time taken to become SARS-CoV-2 PCR-negative after honeysuckle decoction (the day received first dose of HD was recorded as first day); ** Lopinavir/Ritonavir Tablets and Darunavir/Cobicistat Tablets are used in sequence; † WT wild type; ‡ MUT mutant type; § SIDT1, Systemic RNA interference-defective-1 transmembrane family member 1; IVIG, intravenous immunoglobulin.

Table S4 Adverse events (AE) of patients in MIR2911^{+} group and MIR2911^{-} group

AE	MIR2911^{+} group N=6		MIR2911^{-} group N=69	
	any grade	grade 3 or 4	any grade	grade 3 or 4
Syncope	0	0	2	2
Diarrhea	0	0	25	0
Nausea and vomiting	0	0	13	0
Rash maculo-papular	0	0	5	0
Anorexia	0	0	7	0
Alanine aminotransferase increased	1	0	12	1
Aspartate aminotransferase increased	0	0	3	0
Blood bilirubin increased	0	0	3	0
Hypokalemia	0	0	9	0

AE were classified according to the National Cancer Institute Common Terminology Criteria for Adverse Events, version 5.0.
